# A New Method for Biostatistical *miRNA* Pattern Recognition with Topological Properties of Visibility Graphs in 3D Space

**DOI:** 10.1155/2019/4373760

**Published:** 2019-06-11

**Authors:** Matej Babič, Ninoslav Marina, Andrej Mrvar, Kumar Dookhitram, Michele Calì

**Affiliations:** ^1^Faculty of Information Studies, Novo Mesto, Slovenia; ^2^University of Information Science and Technology “St Paul the Apostle”, Skopje, North Macedonia; ^3^Faculty of Social Sciences, University of Ljubljana, Ljubljana, Slovenia; ^4^University of Technology, Mauritius, Port Louis, Mauritius; ^5^Electric, Electronics and Computer Engineering Department, University of Catania, Catania, Italy

## Abstract

Visibility is a very important topic in computer graphics and especially in calculations of global illumination. Visibility determination, the process of deciding which surface can be seen from a certain point, has also problematic applications in biomedical engineering. The problem of visibility computation with mathematical tools can be presented as a visibility network. Instead of utilizing a 2D visibility network or graphs whose construction is well known, in this paper, a new method for the construction of 3D visibility graphs will be proposed. Drawing graphs as nodes connected by links in a 3D space is visually compelling but computationally difficult. Thus, the construction of 3D visibility graphs is highly complex and requires professional computers or supercomputers. A new method for optimizing the algorithm visibility network in a 3D space and a new method for quantifying the complexity of a network in DNA pattern recognition in biomedical engineering have been developed. Statistical methods have been used to calculate the topological properties of a visibility graph in pattern recognition. A new *n*-hyper hybrid method is also used for combining an intelligent neural network system for DNA pattern recognition with the topological properties of visibility networks of a 3D space and for evaluating its prospective use in the prediction of cancer.

## 1. Introduction

Manufacturing the visibility network (graph) [1] is a fundamental geometric structure which has useful applications in several fields including illumination and rendering, motion planning, pattern recognition, and sensor networks. A graph *G* is called a visibility graph when there is a polygon *P* such that the vertices of *P* are the vertices of *G* and two vertices are adjacent in *G* if they are visible in *P*. A visibility graph can be used in spatial analysis of urban and building spaces and applied to landscapes, as well. It is formed by taking a set of points across the space and forming graph edges between those points, if they are mutually visible. Visibility graphs have been widely used for 2D applications so far, but in this paper, an application to complex 3D visibility problems is advanced.

Visibility calculations are central to any computer graphics application. The relevance of statistics [2] has been much more recognized in the biomedical engineering field. Statistics can provide technicians of laboratories with important instruments for a scientific analysis of biomedical phenomena, allowing to understand the observed phenomena more correctly and to obtain more reliable results.

Biomedical engineering [3] being one of the fastest growing engineering disciplines aims at applying engineering expertise and advances to the field of medical needs and bioscience for the enhancement of healthcare.

Deoxyribonucleic acid (*DNA*) pattern recognition constitutes one of the most important works in biomedical engineering. Pattern recognition [4] is a branch of machine learning that focuses on the recognition of patterns and regularities in data, although it is in considered to be in some cases nearly synonymous with machine learning. In pattern recognition, labels are assigned to objects and all objects are described by features, also called attributes. A classic example is the recognition of handwritten digits for the purpose of automatic mail sorting. Pattern recognition methods based on machine learning techniques [5] have been shown to be a promising approach to the analysis of network data. Intelligent computing has attracted many scientists and researchers working on intelligent techniques for solving complex real-world problems.

The graphical representations of the *DNA* as a method for *DNA* pattern recognition have been introduced to facilitate comparison of *DNA* sequences and to observe differences in their structure [6–10]. A novel method is thus explored in this study to generate and characterize complex networks by means of analysis of their *miRNA* sequences.

A new hybrid method [11] that combines three types of intelligent neural network systems [12] has been used. This paper explores the use of an intelligent system [13] with a new hybrid method that improves the existing ones. It is based on the *n*-hyper hybrid method. The aim of this work is to outline possibilities for applying an *n*-hyper hybrid system method for *miRNA* pattern recognition [14] with topological properties of visibility graphs in a 3D space and to evaluate its prospective use in biomedical engineering.

The proposed method can be utilized to approach more systematically problems of visibility of *miRNA* sequences, in the comparison of *DNA* sequences and in the analysis of complex networks such as *miRNA* networks in the biomedical field. The method is based on an algorithm which is built for optimizing the visibility in a 3D space and an *n*-hyper hybrid system for *miRNA* cancer pattern recognition which allows to register more precisely the risk of various types of cancer.

In particular, the proposed method can be used to analyze the transformation of 1D *miRNA* sequences into 3D *miRNA* sequences for predicting cancer. More specifically, a new type of intelligent system, the *n*-hyper hybrid system, has been developed to describe *miRNA* sequences and the difference between cancer and noncancer *miRNA*. Thanks to this method, some more information on a complex network derived from the visibility network in a 3D space, compared to the visibility graph, can be obtained in the application of *miRNA* pattern recognition. The utility of applying 3D visibility graphs in biomedical engineering for a more precise prediction of cancer will be thus discussed in this paper.

Finally, this new method permits to obtain a physical visualization of the three-dimensional *miRNA* sequences. Through additively manufactured (3D printing) techniques, which are much used in the biomedical field for building and analysis of implantable devices [15–18], it is possible indeed to have a reconstruction of the 3D space network as a lattice structure as well as a view of the relationships between *miRNA* sequences.

This paper is organized as follows: Section 2 is devoted to a description of the methods and the materials used. In Section 3, the results and discussions are illustrated. Final considerations and conclusions are drawn in Section 4.

## 2. Materials and Methods

### 2.1. A New Method for Statistical *miRNA* Pattern Recognition

*DNA* [19–23] is composed of an extremely long array of nucleotides. MicroRNAs [23–27] constitute a recently discovered class of noncoding *RNAs* playing some key roles in the regulation of gene expression. In this article, we have developed a new method to describe the transformation of 1D *miRNA* sequences into 3D *miRNA* sequences. This means that we have transformed the *miRNA* sequences into a 3D coordinate system.

**Figure 1:**
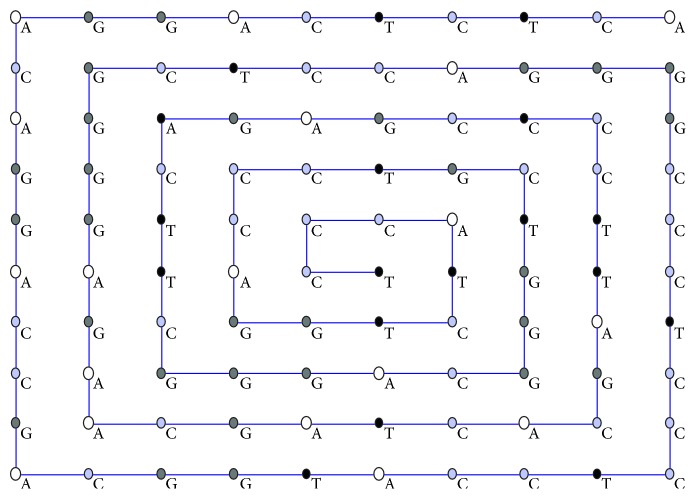
Coloured *miRNA* sequences (miR-612 gene) and transformation of each nucleotide of the *DNA* sequences into a 2D array by using a spiral curve.

For better presentation, four colours are used to denote the nucleotides adenine (A), cytosine (C), guanine (G), and thymine (T). We have replaced each nucleotide by different colours, namely, A by white, C by light purple, G by gray, and T by black. Initially, we have transformed each nucleotide of the *DNA* sequences into a 2D array by using a spiral curve.  Figure 1 presents the coloured *miRNA* sequences (miR-612 gene) and transformation of each nucleotide of the *miRNA* sequences into a 2D array by using a spiral curve. We have also denoted the *miRNA* sequences by two coordinates (*x*, *y*).

**Figure 2:**
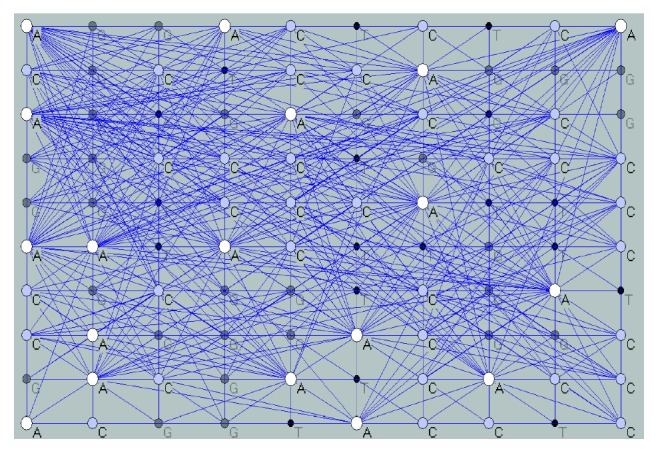
Visibility graph of *miRNA* sequences (miR-612 gene).

Furthermore, we have denoted each nucleotide of the *miRNA* sequences with a number. An example of an *miRNA* sequence is … *TGCCAATCGTTGT* …. This sequence of letters can be converted with the function *f*. We have denoted {*A*, *C*, *G*, *T*} ∈ *M* and {1, 2, 3, 4} ∈ *N*. Also, *f* :*M* ⟶ *N*. We have denoted with *n*_A_, *n*_C_, *n*_G_, and *n*_T_ the number of all nucleotides *A*, *C*, *G*, and *T* in some sequences. We have determined linear arrangement {*n*_A_, *n*_C_, *n*_G_, *n*_T_} from the largest to the smallest value. For example, if *n*_A_ > *n*_C_ > *n*_G_ > *n*_T_, then we have function *f*, *f* : *A* ⟶ 4, *f* :*C* ⟶ 3, *f* : *G* ⟶ 2, and *f* : *T* ⟶ 1 or *f*(*A*) = 4, *f*(*C*) = 3, *f*(*G*) = 2, and *f*(*T*) = 1. When using function *f*, we can write *f*(… *TGCCAATCGTTGT* …) = … 1233441231121 …. Also, the function *f* denotes the third coordinate of the *miRNA* nucleotides in a 3D space, (*x*, *y*, *z*). In the third step, we have used a new method for optimizing the algorithm visibility graph in a 3D space (Figure 2).

For each visibility graph of the *miRNA* sequences, we have calculated the statistical property chi-square of triads [28].

If *ν* independent variables *x*_*i*_ are each normally distributed with mean *µ*_*i*_ and variance *σ*_*i*_^2^, then the quantity known as chi-square (*χ*^2^) is denoted by

(1) $$\begin{array}{c} \chi^{2}=\frac{{\sum \left(x_{i}-\mu_{i}\right)}^{2}}{\sigma_{i}^{2}}. \end{array}$$

### 2.2. A New Method for Optimizing the Algorithm Visibility Graph in 3D Space

We have developed a new algorithm for the construction of visibility graphs in a 3D space [29]. Two arbitrary data values (*x*_*a*_, *y*_*a*_) and (*x*_*b*_, *y*_*b*_) will have visibility and will consequently become two connected nodes of the associated graph if any other data (*x*_*c*_, *y*_*c*_) placed between them fulfil the following relation [30]:

(2) $$\begin{array}{c} y_{c}<y_{b}+\frac{\left(y_{a}-y_{b}\right)\left(x_{b}-x_{c}\right)}{x_{b}-x_{a}}. \end{array}$$

**Figure 3:**
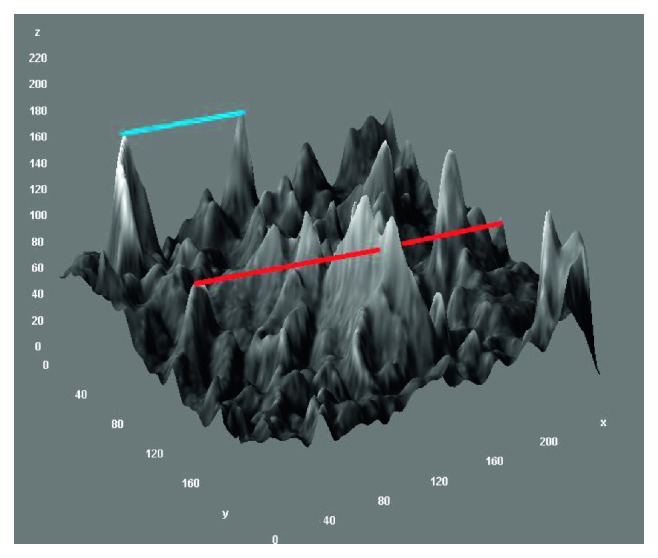
Visibility nodes (blue line) and unrelated nodes (red line).

We wanted to know how to connect the nodes in Figure 3. Nodes *v*_*i*,*j*_ and *v*_*k*,*l*_ of the 3D graph, where *i* < *k* and *j* < *l*, are connected by a link if and only if they are visible.

**Figure 4:**
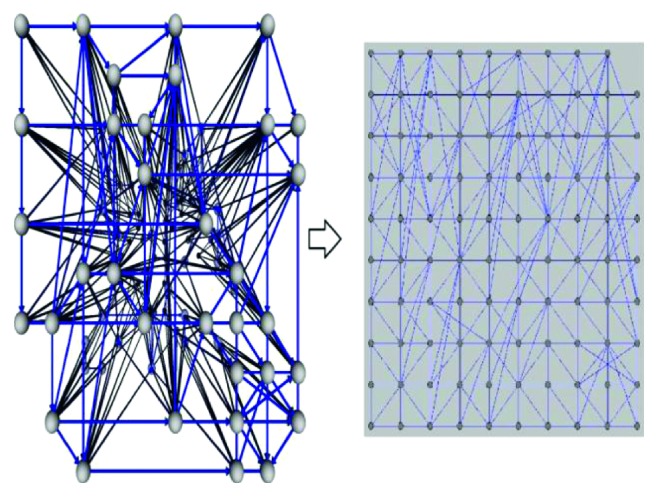
Solution of the 3D visibility graph presented as a 3D graph in a 3D space and in a 2D space.

This means that the path from *v*_*i*,*j*_ to *v*_*k*,*l*_ has no points on the graph. An example is presented in Figure 3, in which the nodnes connected by the blue line are visible to each other and those connected by the red line are an example of unrelated nodes (the straight line that connects the two nodes pierces the graph, which is contrary to the definition of the visibility graph). The following section describes how to construct a 3D visibility graph. The open problem of visibility graphs in a 2D space has thus been presented (Figure 4). Also, we have transformed all 3D points by perpendicular projection on the *xy* plane.

**Figure 5:**
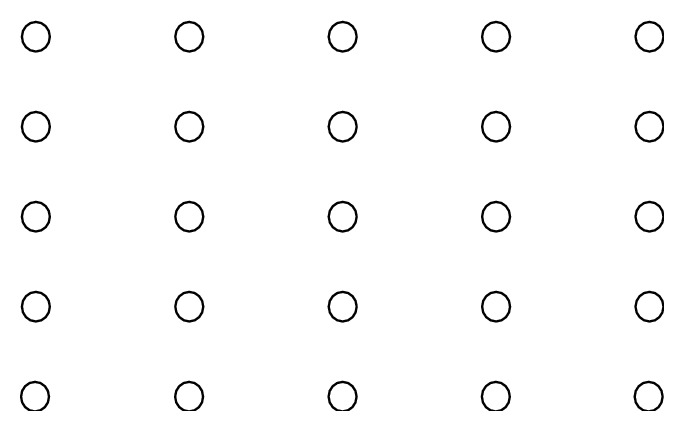
3D nodes which are transformed into a 2D plane.

As a first step, we have the 3D points which are transformed into a 2D plane. A graphical solution on a 5 × 5 grid is given because it provides a better visual representation.  Figure 5 presents the nodes of the graph.

**Figure 6:**
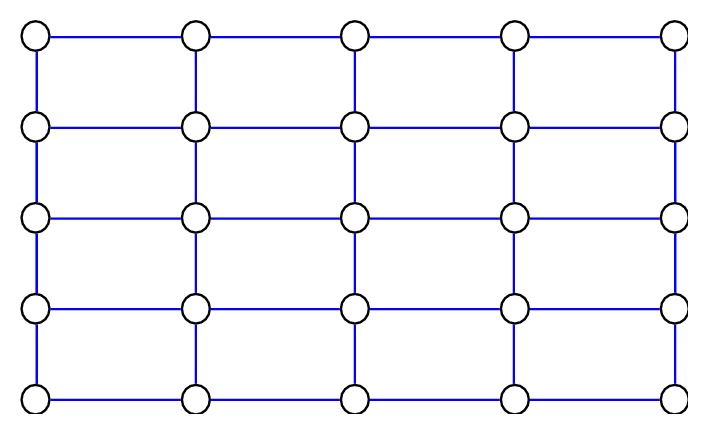
Neighbouring nodes.

In the second step, we have connected neighbouring nodes. Also, node *T*_*i,j*_(*x*_*i*_*, y*_*j*_) is connected with nodes *T*(*x*_*i*−1_, *y*_*j*_), *T*(*x*_*i+1*_, *y*_*j*_), *T*(*x*_*i*_, *y*_*j*+1_), and *T*(*x*_*i*_, *y*_*j*−1_) if node *T*_*i,j*_(*x*_*i*_, *y*_*j*_) is not located on the edge of a complex network. If node *T*_*i,j*_(*x*_*i*_, *y*_*j*_) is on the edge of a complex network, then it is connected with two or three nodes only, as presented in Figure 6.

**Figure 7:**
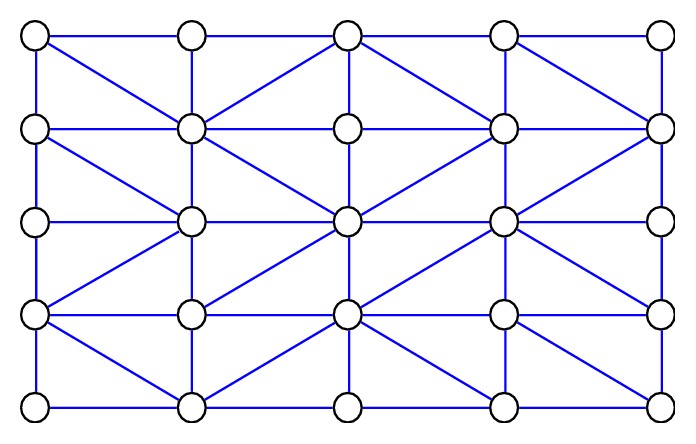
Nodes connected with other nodes by diagonal lines.

In the third step, nodes *T*_*i,j*_(*x*_*i*_, *y*_*j*_), *T*(*x*_*i*+1_, *y*_*j*_), *T*(*x*_*i*+1_, *y*_*j*+1_), and *T*(*x*_*i*_, *y*_*j*+1_, *z*_*i,j*+1_) present quadrilaterals, and it is possible to see many quadrilaterals in Figure 7. In all quadrilaterals, nodes from a higher *z* coordinate are connected with other nodes by diagonal lines.

**Figure 8:**
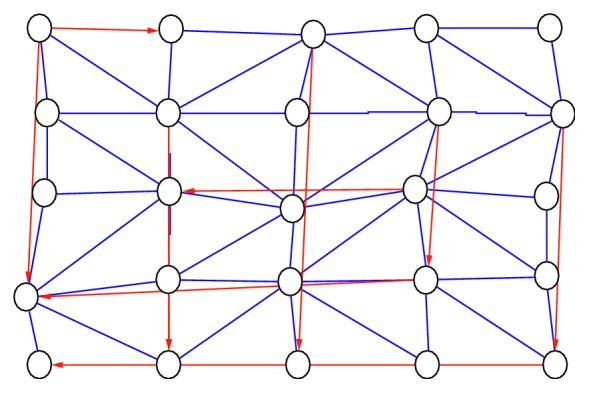
Fourth step of visibility graph creation.

In the fourth step (Figure 8), we have broken the set of all nodes (*x*_*i*_, *y*_*i*_, *z*_*i,j*_) into two sets. The first set presents all nodes (*x*_*i*_, 0, *z*_*i,j*_), and the second set presents all nodes (0, *y*_*i*_, *z*_*i,j*_). We have presented those nodes that are visible in the first set and those that are visible in the second set. In the first set, two nodes *T*_*i,j*_(*x*_*i*_, *y*_*j*_, *z*_*i,j*_) and *T*_*k,l*_(*x*_*k*_, *y*_*l*_, *z*_*k,l*_) are visible and will consequently become connected nodes on the associated graph if any other node *T*(*x*_*m*_, 0, *z*_*m*,0_) placed between them fulfils the following relation:

(3) $$\begin{array}{c} z_{m,0}<z_{k,l}+\frac{\left(z_{i,j}-z_{k,l}\right)\left(x_{k}-x_{m}\right)}{\left(x_{k}-x_{i}\right)}. \end{array}$$

In the second set, two nodes *T*_*i,j*_(*x*_*i*_, *y*_*j*_, *z*_*i,j*_) and *T*_*k,l*_(*x*_*k*_, *y*_*l*_, *z*_*k,l*_) are visible and will consequently become two connected nodes on the associated graph if any other node *T*_0,*n*_(0, *y*_*n*_, *z*_0,*n*_) placed between them fulfils the following relation:

(4) $$\begin{array}{c} z_{m,0}<z_{k,l}+\frac{\left(z_{i,j}-z_{k,l}\right)\left(y_{k}-y_{m}\right)}{\left(y_{k}-y_{i}\right)}. \end{array}$$

**Figure 9:**
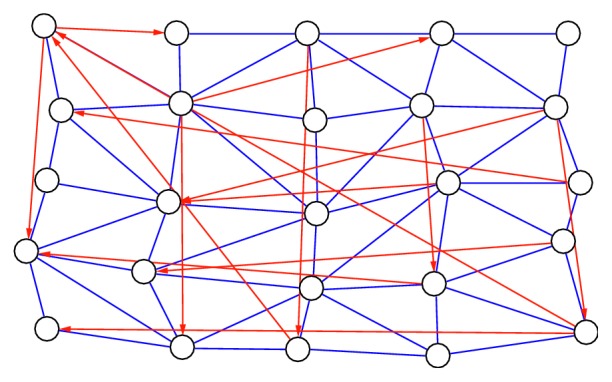
Solution of the 3D visibility graph on a 5 × 5 grid.

In the fifth step, we have connected all other visible nodes. Two nodes *T*_*i,j*_(*x*_*i*_, *y*_*j*_, *z*_*i,j*_) and *T*_*k,l*_(*x*_*k*_, *y*_*l*_, *z*_*k,l*_) will be visible if no nodes exist on the line between *T*_*i,j*_(*x*_*i*_, *y*_*j*_, *z*_*i,j*_) and *T*_*k,l*_(*x*_*k*_, *y*_*l*_, *z*_*k,l*_).  Figure 9 presents the solution of the 3D visibility graph on a 5 × 5 grid.

### 2.3. Optimizing the Algorithm Visibility Network in 3D Space

- All 3D points transform into 2D points {(*x*, *y*, *z*) ⟶ (*x*, *y*)}
- Node *T*_*i,j*_(*x*_*i*_, *y*_*j*_) is connected with nodes *T*(*x*_*i*−1_, *y*_*j*_), *T*(*x*_*i*+1_, *y*_*j*_), *T*(*x*_*i*_, *y*_*j*+1_), and *T*(*x*_*i*_, *y*_*j*−1_), if node *T*_*i,j*_(*x*_*i*_, *y*_*j*_) is not located on the edge of a complex network
- If node *T*_*i,j*_(*x*_*i*_, *y*_*j*_) is on the edge of a complex network, then it is connected with two or three nodes only
- Nodes from a higher *z* coordinate of all nodes *T*_*i,j*_(*x*_*i*_, *y*_*j*_)*, T*(*x*_*i*+1_, *y*_*j*_)*, T*(*x*_*i*+1_, *y*_*j*+1_), and *T*(*x*_*i*_, *y*_*j*+1_, *z*_*i,j*+1_) in quadrilaterals are connected with other nodes by diagonal lines
- Two nodes *T*_*i,j*_(*x*_*i*_, *y*_*j*_, *z*_*i,j*_) and *T*_*k,l*_ (*x*_*k*_, *y*_*l*_, z_*k,l*_) are connected, if any other node *T*(*x*_*m*_, 0, *z*_*m*,0_) placed between them fulfils *z*_*m*,0_ < *z*_*k,l*_ + (*z*_*i,j*_ − *z*_*k,l*_)(*x*_*k*_ − *x*_*m*_)/(*x*_*k*_ − *x*_*i*_)
- Two nodes *T*_*i,j*_(*x*_*i*_, *y*_*j*_, *z*_*i,j*_) and *T*_*k,l*_(*x*_*k*_, *y*_*l*_, *z*_*k,l*_) are connected, if any other node T_0,*n*_(0, *y*_*n*_, *z*_0,*n*_) placed between them fulfils *z*_*m*,0_ < *z*_*k,l*_ + (*z*_*i,j*_ − *z*_*k,l*_)(*y*_*k*_ − *y*_m_)/(*y*_*k*_ − *y*_*i*_)
- Two nodes *T*_*i,j*_(*x*_*i*_, *y*_*j*_, *z*_*i,j*_) and *T*_*k,l*_(*x*_*k*_, *y*_*l*_, *z*_*k,l*_) are connected, if no nodes exist on the line between *T*_*i,j*_(*x*_*i*_, *y*_*j*_, *z*_*i,j*_) and *T*_*k,l*_(*x*_*k*_, *y*_*l*_, *z*_*k,l*_)

### 2.4. A New *n*-Hyper Hybrid Method

Hybrid evolutionary computation is a generic, flexible, robust, and versatile method for solving complex global optimization problems and can also be used in practical applications. Only three methods are adopted using intelligent systems, namely, the neural networks NN1 (present prediction with 33%; also, we use 67% of the data for the learning set and 33% for the test set), NN2 (present prediction with 50%; also, we use 50% of the data for the learning set and 50% for the test set), and NN3 (present leave-one-out cross-validation method). We have used a four-layer network with a learning rate of 0.7, moment of learning of 0.6, tolerance of test set of 0.02, and tolerance of learning set of 0.2. Hybrid 1 presents the sequence hybrid method. In this hybrid, methods are connected in series in the direction of the entrance to the method *n*. All methods work independently of the other methods. The results of input method 1 are transferred to input method 2, and the results of input method 2 are transferred to input method 3. Hybrid 2 presents the cyclic hybrid method. In Hybrid 2, methods are connected in series in the direction of the entrance to the method *n*. All methods work independently of the other methods.

**Figure 10:**
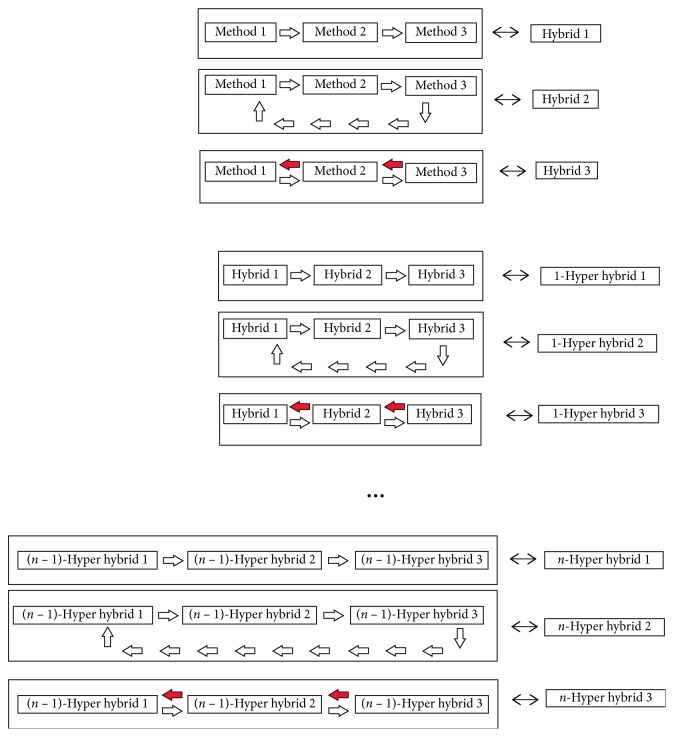
*n*-Hyper hybrid method.

The results of input method 1 are transferred to input method 2, the results of input method 2 are transferred to input method 3, and the results of input method 3 are transferred to input method 1. In Hybrid 3, all methods work independently of the other methods. The results of input method 1 are transferred to input method 2, the results of input method 2 are transferred to input method 3, the results of input method 3 are transferred to input method 2, and the results of input method 2 are transferred to input method 1. 1-hyper hybrid methods are similar to hybrid methods. Also, we have repeated the process up to *n*, where *n* ∈ *N*. In the end, we have *n*-hyper hybrid methods.  Figure 10 presents all the processes of building *n*-hyper hybrids.

## 3. Results and Discussion

The visibility graph problem itself has long been studied in computational geometry and has been applied to a variety of areas. We present a new method for describing the transformation of 1D *miRNA* sequences into 3D *miRNA* sequences. We combine this method with a new method for optimizing the algorithm visibility graph in a 3D space. Based on the variation network, several topological properties, such as different types of triads, are calculated for natural *miRNA* sequences. Also, the correlations between types of triads over the variation network are obtained.

It is well known that there is an individual cancer susceptibility despite equivalent environmental exposure, likely due to polymorphisms in genes involved in carcinogenesis.  Table 1 presents a list of *miRNA* gene polymorphisms associated with cancer. We use the miR-146a, hsa-mir-149, hsa-mir-196a-2, hsa-mir-608, and hsa-miR-612 genes from the *miRNA* base. MiR-146a is a family of microRNA precursors found in mammals, including humans. MiR-146a is primarily involved in the regulation of inflammation and other processes that function in the innate immune system. Loss of functional miR-146a (and mir-145) could predispose an individual to suffer from chromosome 5q deletion syndrome. MiR-146 has also been reported to be highly upregulated in the osteoarthritis cartilage and could be involved in its pathogenesis. An increasing body of evidence points to a possible role of microRNAs (*miRNAs*) in hereditary cancer syndromes [31, 32].

Recently, variations of the miR-146a gene have drawn increasing attention in cancer etiologies, and altered expression levels have been observed in inflammatory diseases as well as in cancers [33, 34]. MicroRNA hsa-mir-149 is located on chromosome 2. It is an intronic *miRNA* and is located in sense orientation relative to its protein-coding host gene glypican 1 (*GPC1*). Our integrated review of *miRNA*-SNPs revealed that polymorphisms of hsa-mir-149 have previously been associated with increased or decreased risk of seven cancer types: renal cell carcinoma and breast, colorectal, gastric, hepatocellular, papillary, and thyroid cancers. miR149 rs71428439 predisposes its carriers to CCRCC, and miR149 rs71428439 may be a new biomarker for predicting the risk of CCRCC [35]. miR-196 appears to be a vertebrate-specific microRNA and has now been predicted or experimentally confirmed in a wide range of vertebrate species (MIPF0000031). The hairpin precursors are predicted based on base pairing and cross-species conservation—their extents are not known. Many studies demonstrated that the hsa-miR-196a2 rs11614913 SNP was significantly associated with the susceptibility of breast cancer [36–38]. MicroRNA hsa-mir-608 is located on chromosome 10. It is an intronic *miRNA*, located in sense orientation relative to its host gene semaphorin 4G (*SEMA4G*) and in antisense orientation to the mitochondrial ribosomal protein *L43M* (*RPL43*) gene. Several studies [39–46] examined the impact of miR-608 rs4919510C>G on the risk of various cancers, but the results were inconsistent.

Additionally, it has also been associated with increased risk of breast, nasopharyngeal, and papillary thyroid carcinomas. MicroRNA hsa-mir-612 is located on chromosome 11. It is an exonic *miRNA*, located in sense orientation relative to its host gene, nuclear paraspeckle assembly transcript 1 (*NEAT1*). Polymorphism with pre-*miRNA* regions has been associated with B-cell acute lymphoblastic leukemia [47–49].

Also, we have used a new method for optimizing the algorithm visibility graph in a 3D space and a new method for *miRNA* cancer pattern recognition. We have determined the topological properties of the triad patterns, in *miRNA* networks [50]. The analysis of triads and the prevalence of different types of triads in populations has been a staple of most network analyses. The input data of neural networks are the topological properties, namely, the triads of *miRNA* patterns and function *f* of sequences of *miRNA*. The output data (*Y*) of the neural data decide whether the *miRNA* is associated with cancer: if the *miRNA* is not associated with cancer, then 1 is output, else 0 is output.

Table 2 presents the topological properties of the visibility graphs in a 3D space of *DNA* patterns. D1–D5 present the mark of *DNA*. C1–C5 present cancer D1–D5 *DNA*. TP1–TP10 [51] present the types of topological properties of triads: TP1 presents type 1-102, TP2 presents type 1-003, TP3 presents type 2-012, TP4 presents type 6-021C, TP5 presents type 7-111D, and TP6 presents type 8-111U. For each constructed variation *miRNA* network, the related topological properties are shown in Table 2. We can see that triad TP1 type 1-102 increases for cancer *miRNA* (rows C1–C5). In the third column, we can see that triad TP3 type 2-012 decreases for cancer *miRNA* (rows C1–C5). In the fourth column, we can see that triad TP4 type 6-021C decreases for cancer *miRNA* (rows C1–C5). In the fifth column, we can see that triad TP6 type 8-111U decreases for cancer *miRNA* (rows C1–C5). Also, these three types of triads present a significant correlation between *miRNA* and cancer *miRNA*.

Table 3 presents the statistical properties of the graph of *miRNA* patterns. *X* presents the modification of the nucleotide position. S1 presents the place number of changed nucleotides in *miRNA*. S2 presents the number of all edges in the graph. S3 presents the chi-square of triads. In hsa-mir-146a, nucleotide A changes into G on the 50th place, which is described in column S1. The chi-square of the triads increases on hsa-mir-149, hsa-mir-196a-2, and hsa-mir-608 in another decrease.

Table 4 presents the experimental and predicted cancers of *miRNA* patterns. Column C presents the decision of cancer *miRNA* with different methods. In Table 4, rows NN1, NN2, and NN3 present the data predicted with the neural network, rows H1, H2, and H3 present the data predicted with the hybrid system, 1-H1, 1-H2, and 1-H3 present the data predicted with the 1-hyper hybrid system, 2-H1, 2-H2, and 2-H3 present the data predicted with the 2-hyper hybrid system, and 10-H1, 10-H2, and 10-H3 present the data predicted with the 10-hyper hybrid system.

The first row is equal to column *Y* in Table 2, which determines whether the *miRNA* is cancer or noncancer *miRNA*. Modeling with NN1 presents a 30% precision from the set of measured data, modeling with NN2 presents a 60% precision from the set of measured data, modeling with NN3 presents a 70% precision from the set of measured data, modeling with H1 presents a 30% precision from the set of measured data, modeling with H2 presents a 30% precision from the set of measured data, modeling with H3 presents a 30% precision from the set of measured data, modeling with 1-HH1 presents a 30% precision from the set of measured data, modeling with 1-HH2 presents a 50% precision from the set of measured data, modeling with 1-HH3 presents a 60% precision from the set of measured data, modeling with 2-HH1 presents a 30% precision from the set of measured data, modeling with 2-HH2 presents a 50% precision from the set of measured data, modeling with 2-HH3 presents a 70% precision from the set of measured data, modeling with 10-HH1 presents a 50% precision from the set of measured data, modeling with 10-HH2 presents a 70% precision from the set of measured data, and modeling with 10-HH3 presents a 90% precision from the set of measured data. Therefore, we can see that 10-HH3 presents the best prediction.

## 4. Conclusions

In this paper, a novel concept entitled “optimizing the algorithm visibility graph in a 3D space network” is introduced to analyze the relationships between *miRNA* sequences and the type of cancer *miRNA*. We have developed a new method for describing the transformation of 1D *miRNA* sequences into 3D *miRNA* sequences. Using the topological properties of different types of triads, we have determined *miRNA* sequences and the difference between cancer and noncancer *miRNA*. From the results obtained, we are able to conclude that the variation network is a complex network and that it has some dynamic information for further researches. The visibility network in a 3D space, which contains more information than the visibility graph, has been used for the application of *miRNA* pattern recognition in biomedical engineering. This new method permits to obtain also a physical visualization of the tridimensional *miRNA* sequences through additively manufactured techniques.

Finally, we have built a new type of intelligent system, the *n*-hyper hybrid system, that can be used for cancer *miRNA* prediction.

## Figures and Tables

**Table 1:** List of *miRNA* gene polymorphisms associated with cancer.

*miRNA* name	Mark	rs number	Nucleotide change	Cancer type
hsa-mir-146a	D1	2910164	A>G	Increased risk for gastric cancer
hsa-mir-149	D2	71428439	A>G	Increased risk for chronic lymphocytic leukemia
hsa-mir-196a-2	D3	11614913	C>T	Breast cancer
hsa-mir-608	D4	4919510	C>G	Increased risk for breast cancer
hsa-mir-612	D5	12803915	G>A	B-cell acute lymphoblastic leukemia

**Table 2:** Topological properties of the graph of *miRNA* patterns.

*N*	TP1	TP2	TP3	TP4	TP5	TP6	*Y*
D1	15359	126096	17484	489	505	522	0
C1	15372	126020	17446	486	509	525	1
D2	15511	126406	17240	497	514	469	0
C2	15611	126482	17082	490	509	470	1
D3	18875	167586	20947	424	530	522	0
C3	18886	167784	20753	421	524	525	1
D4	15601	125597	18093	402	488	480	0
C4	15603	125596	18085	401	487	481	1
D5	15611	126952	16657	454	493	469	0
C5	15639	126953	16655	453	492	473	1

**Table 3:** Statistical properties of the graph of *miRNA* patterns.

*N*	*X*	S1	S2	S3
D1	A	50	395	251228
C1	G	50	396	249883
D2	A	83	392	265112
C2	G	83	390	269117
D3	C	78	425	365987
C3	T	78	423	368945
D4	C	37	399	265911
C4	G	37	399	266532
D5	G	51	386	273025
C5	A	51	386	272976

**Table 4:** Experimental and predicted cancers of *miRNA* patterns.

*N*	D1	C1	D2	C2	D3	C3	C4	D4	C5	D5
E	0	1	0	1	0	1	0	1	0	1
NN1	1	0	0	0	0	1	1	0	1	0
NN2	1	1	1	1	0	0	0	1	1	1
NN3	0	1	0	1	0	0	0	1	1	1
H1	0	0	1	0	1	1	1	0	1	0
H2	1	1	1	1	1	1	0	1	1	0
H3	0	1	0	0	0	0	1	1	1	1
1-HH1	1	0	1	0	1	1	1	0	0	0
1-HH2	0	1	1	0	0	0	0	1	1	0
1-HH3	1	1	0	1	0	0	0	0	0	1
2-HH1	1	0	0	0	1	1	1	1	1	0
2-HH2	1	1	1	1	1	1	1	0	0	1
2-HH3	0	1	0	0	0	0	0	1	0	1
10-HH1	1	0	0	1	1	1	1	1	1	1
10-HH2	1	0	0	1	0	0	0	1	0	1
10-HH3	1	1	0	1	0	1	0	1	0	1

## Data Availability

The data in Table 1 are available online at http://www.ncbi.nlm.nih.gov/pmc/articles/PMC4586796/.
